# Incidence, risk factors and impact on outcomes of secondary infection in patients with septic shock: an 8-year retrospective study

**DOI:** 10.1038/srep38361

**Published:** 2016-12-07

**Authors:** Guang-ju Zhao, Dong Li, Qian Zhao, Jia-xing Song, Xiao-rong Chen, Guang-liang Hong, Meng-fang Li, Bing Wu, Zhong-qiu Lu

**Affiliations:** 1 Emergency Department, The First Affiliated Hospital of Wenzhou Medical University, Wenzhou 325000, P.R.China

## Abstract

Secondary infection in septic patients has received widespread attention, although clinical data are still lacking. The present study was performed on 476 patients with septic shock. Time trends for mortality were analyzed using Spearman’s rank correlation test. Risk factors for secondary infection were investigated by binary logistic regression. The extended Cox model with time-varying covariates and hazard ratios (HR) was performed to determine the impact of secondary infection on mortality. Differences in hospital length of stay (LOS) between patients with and without secondary infection were calculated using a multistate model. Thirty-nine percent of septic shock patients who survived the early phase of the disease developed secondary infection. There was a statistically significant increased odds ratio for secondary infection in older patients and patients with a longer LOS in the intensive care unit (ICU), a higher Sequential Organ Failure Assessment (SOFA) score, and endotracheal intubation. Secondary infection significantly reduced the rate of discharge (HR 5.607; CI_95_ 3.612–8.704; P < 0.001) and was associated with an increased hospital LOS of 5.46 days. The present findings represent a direct description of secondary infection in septic shock patients and highlight the influence of this condition on septic shock outcomes.

Sepsis, as a major challenge in intensive care and emergency medicine, is typically defined as a hyperinflammatory response resulting from microbial infection^1^. However, many recent studies have demonstrated that sepsis is associated with only a transient hyper-inflammatory phase^2^,^3^,^4^. Subsequently, patients enter a prolonged immunosuppressive phase that is characterized by deficient immune cell responses, apoptotic depletion of immune cells, and an increased percentage of immune-suppressing cells and elevated levels of anti-inflammatory cytokines^2^,^3^,^4^,^5^. As a consequence, many septic patients are at risk for secondary infection, as demonstrated in many clinical studies^6^,^7^,^8^,^9^.

Secondary infection after septic shock has received widespread attention, although clinical data are lacking. In particular, few retrospective studies have been designed to investigate the incidence and impact of secondary infection on clinical outcomes in septic patients. Otto *et al*.^10^ demonstrated that the rates of common opportunistic bacteria and fungi increased significantly in the late phase (>15 days) of severe sepsis and septic shock when compared with the early phase (<6 days) of the disease. Further, another retrospective study found that septic shock patients who died more than 3 days after intensive care unit (ICU) admission were related to ICU-acquired complications, including secondary infections^11^. In contrast, evidence provided by Goldenberg *et al*. illustrated that only 14% of patients who died of septic shock had a new infection at the time of death^12^.

The aims of the present study were to evaluate the incidence and risk factors of secondary infection in patients with septic shock. Furthermore, the impact of secondary infection on septic shock outcomes was also examined.

## Results

### Septic shock patient characteristics

**Figure 1:**
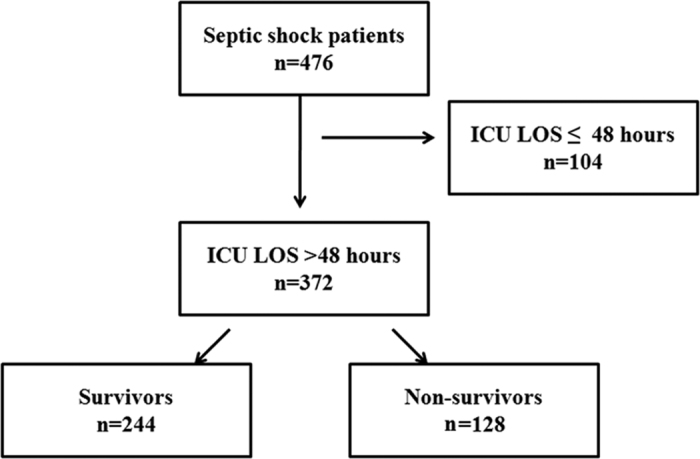
A flow chart of the studied population. A total of 476 septic shock patients met the criteria and were included in the study to assess the time trends of mortality. Three hundred and seventy-two patients with an ICU LOS > 48 hours were included in the assessment of the incidence, risk factors and impact of secondary infection on outcomes. LOS: length of stay.

A total of 476 septic shock patients met the criteria and were included in the study (Fig. 1). The demographic and clinical characteristics of these patients are shown in Table 1. The median age of the patients was 64 (interquartile range [IQR], 53–75) years, and 59.9%were male (n = 285). The major sites of infection were the abdomen (42.4%), followed by the respiratory tract (25.8%), urinary tract (13.2%), skin and soft tissue (12.6%) and other sites of infection (5.9%). The median values of the Simplified Acute Physiology Score II (SAPS II) and Sequential Organ Failure Assessment (SOFA) scores at the onset of septic shock were 48 (IQR 42–56) and 10.00 (7, 12), respectively.

**Figure 2:**
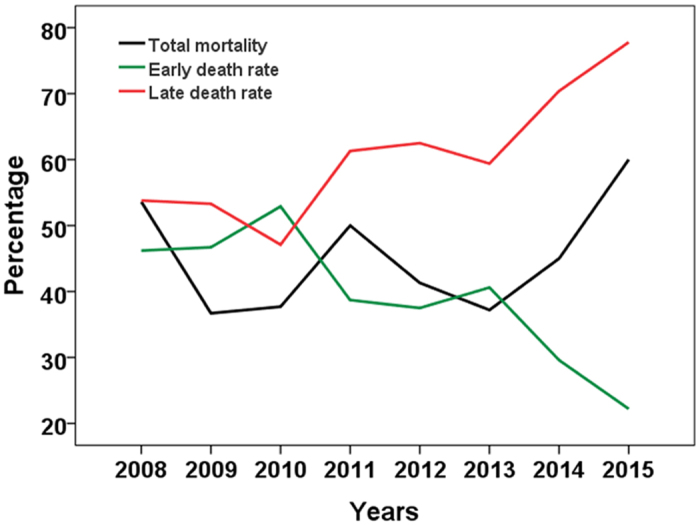
Time trends for total mortality as well as early and late death rates from 2008 to 2015.

The median length of ICU stay and hospital stay were 6 (IQR 3–11) and 12 (IQR 6–21) days, respectively. The ICU and hospital morality were 41.0% (195/476) and 42.8% (204/476), respectively. The peak of death was seen on the second day after ICU admission (see Supplementary Fig. S1). Accordingly, two phases of septic shock were established: the early phase (≤2 days) and the late phase (>2 days). The number of patients who died in the early phase of the disease fell significantly from 2008 to 2015 (r = −0.833, p = 0.010), while the total mortality did not change significantly (Fig. 2).

### Characteristics of secondary infection in septic shock patients

Thirty-nine percent (145/372) of patients who survived the early phase of the disease developed secondary infections in the ICU. Of these, 112 patients had a secondary infection at one site, 29 patients had secondary infections at two sites and 4 patients had three or more secondary infection sites. The median time of the first diagnosis of secondary infection was 9 days (IQR 6–12) (see Supplementary Fig. S2). Among 183 secondary infections, pulmonary infection (PI) was the most frequent secondary infection (52.5%), followed by bloodstream infection (BSI) (23.0%), surgical site and soft tissue infections (SSI/STI) (11.5%), urinary tract infection (UTI) (8.7%) and others (4.3%). Of all secondary infections, 91.3% (167/183) were microbiologically confirmed, and 221 microorganisms were isolated. The most frequently isolated PI microorganism was *Acinetobacter baumannii* (22.2%), followed by *Pseudomonas aeruginosa* (10.3%), and *Stenotrophomonas maltophilia* and *Candida albicans* (both 8.5%). BSIs were mostly caused by *Staphylococcus spp.*, *Candida albicans* and *Enterococcus faecium*. *Pseudomonas aeruginosa* and *Enterococcus faecium* accounted for 48.1% of all microorganisms from SSI/STIs. Additionally, *Candida albicans* was the most common pathogen responsible for UTIs. Details regarding the frequency of isolated microorganisms are given in Table 2.

### Risk factors for secondary infection in septic shock patients

In the univariate analysis, there were statistically significant differences in age, the percentage of patients more than 65 years old, admission category, the SAPS II and SOFA scores, the duration of shock and the length of ICU stay between patients with and without secondary infection (all p < 0.05) (Table 3). Additionally, statistically significant differences were also observed across interventions, including corticosteroid treatment, blood transfusion, renal replacement therapy, and endotracheal intubation (all p < 0.05) (Table 3). The variables mentioned above were selected for multiple logistic regression. In this model, there was a statistically significant increased odds ratio for secondary infection in older patients (odds ratio [OR] 1.020; 95% confidence interval [CI_95_] 1.004~1.036, p = 0.016) and those with a longer LOS in the ICU (OR 1.070; CI_95_ 1.036~1.105, p < 0.001), a higher SOFA score (OR 1.117; CI_95_ 1.040~1.200, p = 0.002), and endotracheal intubation (OR 2.462; CI_95_ 1.492~4.061, p < 0.001)(Table 4).

### Impact of secondary infection on hospital death of septic shock patients

**Figure 3:**
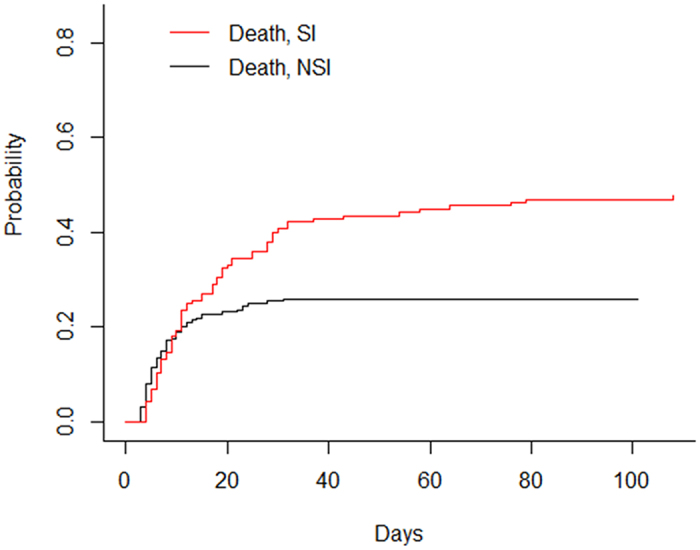
Cumulative incidence functions for discharge and death. Solid lines: discharge; dashed lines: death; read lines: secondary infection; black lines: no secondary infection. SI: secondary infection; NSI: no secondary infection.

As shown in Table 3, the ICU and hospital mortality of patients with secondary infection were 42.8% and 47.6%, respectively. In patients without secondary, the ICU and hospital mortality were 25.6% and 26.0%, respectively. The difference of hospital and ICU mortality were observed between patients with and without secondary infection (P < 0.001 and P = 0.001, respectively). To further investigate the impact of secondary infection on hospital death of septic shock patients, Cox proportional hazards modeling was used with secondary infection modeled as a time-varying covariate. Unadjusted and adjusted hospital mortality hazard ratios for SI versus non-SI were shown in Table 5. The risk of hospital death for patients with SI was 5.7 times higher than that for patients who remained free of SI (HR 5.675; CI_95_ 3.652–8.819; P < 0.001). After adjustment for confounders, including age, site of infection, positive blood culture, SOFA score and duration of shock, the relative risk of hospital mortality, associated with SI, decreased to 5.607 (CI_95_ 3.612–8.704; P < 0.001). Cumulative incidence functions for death were shown in Fig. 3. The cumulative probability of hospital death was greater for an infected patient after around day 10.

### The impact of secondary infection on hospital LOS in septic shock patients

**Figure 4:**
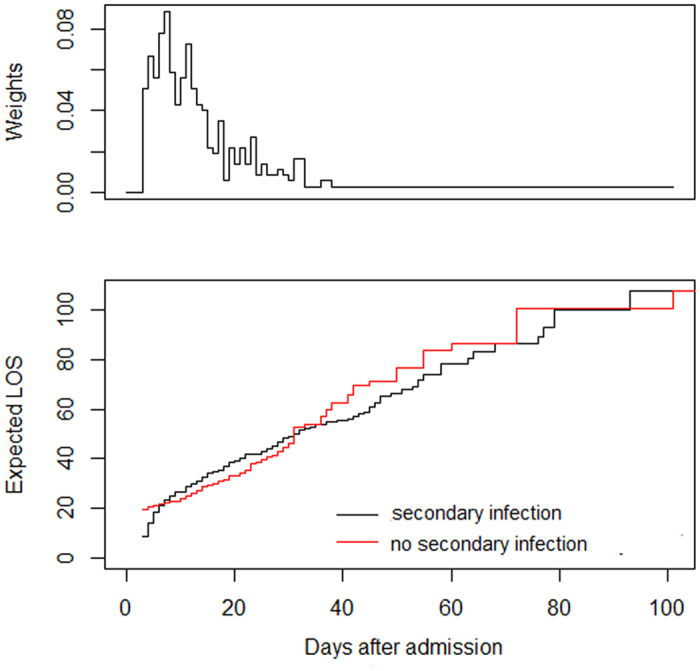
Extra hospital length of stay in patients with (red line) and without (black line) secondary infection. LOS: length of stay.

In the univariate analyses, the median hospital LOSs for patients with and without secondary infection were 13 (IQR 8–20) and 19 (IQR 11–32), respectively (p < 0.001) (Table 3). Other variables associated with LOS were alcohol abuse, skin and soft tissue infection, the duration of shock, SOFA score and steroid treatment (all p < 0.05) (see Supplementary Table S2). Using a multistate model, the expected extra hospital LOS due to secondary infection was 5.46 days based on a standard error of 3.42 days. Patient with secondary infection was clearly observed to have a longer hospital stay between 5 to 30 days after admission (Fig. 4). Additionally, as shown in the weight panel, secondary infections also occurred most frequently in this time period (Fig. 4).

## Discussion

Sepsis is an infection-induced systemic inflammatory response with an estimated mortality of 25%, which can reach up to 60% when shock is present^13^,^14^.Therefore, numerous therapeutic strategies aimed at reducing mortality in these patients have been developed. The first Surviving Sepsis Campaign (SSC) guidelines for the management of severe sepsis and septic shock were published in 2004 and have been updated every four years^15^,^16^,^17^. Thanks to these efforts, mortality resulting from this disease has decreased year by year. For instance, a study with more than one hundred thousand severe sepsis patients illustrated that the absolute morality in severe sepsis patients decreased from 35% to 18.4%^13^. Similar results were also reported by meta-analysis^18^. In the present study, however, the hospital mortality of patients with septic shock did not decrease during the study period. The sustained high mortality from septic shock maybe due to increased mortality in the late phase of the disease, because the mortality fell significantly in the early phase.

There are several factors associated with the death of septic shock patients, including demographic characteristics, the severity of the disease and the therapeutic strategies^19^,^20^. Nevertheless, the risk factors related to late death among septic shock patients remain unclear. Recently, a prospective observational study demonstrated that ICU acquired infections in patients with sepsis contributed modestly to overall mortality^21^. Additionally, a retrospective study illustrated that early deaths were mainly attributable to intractable multiple organ failure, while secondary infection was the second leading cause of late death among patients with severe sepsis and septic shock^11^. Similarly, in present study, we found that the risk of late death for septic shock patients with secondary infection was about 5.8 times higher than that for patients who remained free of secondary infection.

The impact of secondary infection on patients’ LOS has been well documented. In a study of 778 ICU patients, nosocomial infection increased the LOS by 18.2 days per patient^22^. It should be noted that secondary infection can only impact LOS after it has started, and it has been suggested that the duration of hospitalization prior to the infection should be controlled^23^. So, the duration of hospitalization prior to infection should be considered in the analysis of the impact of secondary infection on LOS. Multistate modelling represents a suitable method to avoid time-dependent bias that offering a more precise estimation of extra LOS attributable to hospital-acquired infections^23^. In present study, using a multistate model, we found that patients who suffered secondary infection between 5 to 30 days after admission have a longer hospital stay when compared with non-infected patients. Interestingly, secondary infections also occurred most frequently in this time period and led to a prolonged hospital LOS of 5.46 day.

The unique immune status of sepsis patients may influence their susceptibility to secondary infection. It has been reported that splenocytes and circulating immune cells from sepsis patients show highly significant functional impairment, demonstrated by significantly reduced cytokine secretion^24^,^25^,^26^. Additionally, many sepsis patients who die after 3 days present signs of opportunistic infections and show down-regulated monocytic HLA-DR expression and cytokine production in response to lipopolysaccharide (LPS) stimulation^27^. In present study, the secondary infection rate in patients with septic shock was 39.0%, which was much higher than the rates observed among general ICU patients in mainland China and industrialized countries^28^,^29^. As a retrospective study, the immune status of our patients was not tested. However, we found that LOS in the ICU was positively associated with secondary infection in septic shock patients. Patients who stay longer in ICU are at greater risk of infection may due to the ICU environment itself harbors pathogenic microorganisms^30^. An alternative explanation is that factors associated with nosocomial infection, including underlying conditions and increased use of invasive procedures, may induce a longer ICU stay^31^. In present stay, age, the SOFA score, and endotracheal intubation were observed to be associated with secondary infection. When there remains no reliable strategy for immunomodulatory therapy, the information provided in our study may be useful for reducing or preventing secondary infections after septic shock.

The present study has some limitations. First, differences in treatment and nursing protocols may have influenced the outcomes of patients with and without secondary infection. Nevertheless, all patients who met the criteria within the study period were included in the study, and the potential factors that may be associated with secondary infections and outcomes were recorded. Second, approximately 40% of our patients suffered primary abdominal infection. Therefore, our results may be difficult to duplicate in other studies with low proportions of abdominal infection. Third, it is unknown whether the increased risk of death in patients with septic shock who experience late death is due to the development of secondary infections or whether secondary infections may be an independent risk factor for increased mortality in patients who have a prolonged ICU stay. Lastly, the duration of hospitalization prior to infection was considered in the analysis of the impact of secondary infection on LOS. However, it should be noted that many efforts, such as antibiotic treatment, nursing management and surgery, to prevent a secondary infection may well begin before the diagnostic test is performed and the results are obtained, as described by Mauldin PD *et al*.^32^. This suggests that our assessment of the impact of secondary on LOS should be regarded as a lower estimate. Additionally, as nosocomial infection is likely not unique to patients with septic shock, the impact of it on other types of shock and other ICU admissions need to be further investigated.

## Materials and Methods

### Patients and setting

This retrospective study followed the tenets of the Declaration of Helsinki for research involving human subjects, and the study was reviewed and approved by the Institutional Review Board of the First Affiliated Hospital of Wenzhou Medical University, Wenzhou, China. Because the present study was an observational, retrospective study and all data have been anonymized, informed consent was waived by the Medical Ethics Committee.

The First Affiliated Hospital of Wenzhou Medical University is an adult comprehensive tertiary teaching hospital in Wenzhou, Zhejiang province, P.R. China. There are currently 3300 beds in the hospital. The study took place in 2 mixed ICUs and 1 medical ICU with a total of 95 beds in the hospital. From September 2008 to June 2015, ICU patients over 18 years old with septic shock were enrolled. Because it is difficult to discriminate the pathogens of primary ICU-acquired sepsis and those of secondary infection, patients diagnosed with septic shock after 48 hours of ICU admission were excluded. The diagnosis of septic shock was based on the criteria of the American College of Chest Physicians/Society of Critical Care Medicine (ACCP/SCCM)^33^, as follows: 1) an identifiable site of infection and at least two of the signs of systemic inflammatory response syndrome (SIRS); and 2) arterial blood pressure of <90 mmHg despite adequate fluid resuscitation and requiring vasopressor therapy. Patients were treated according to the strategy described in the 2008 or 2012 Surviving Sepsis Campaign Guidelines^15^,^16^.

### Data collection

#### Baseline data

Baseline data collection included demographics (age and gender), comorbidities, immunosuppressive drugs, and admission category (medical or surgical needs).

#### Characteristics of the primary infection

The primary site of infection, pathogens isolated and the duration of septic shock (the time between the start and stop of vasopressive therapy) were recorded. We also recorded the SAPS II score at the onset of septic shock and the SOFA score at the first 24 hours after the diagnosis of septic shock.

#### Interventions

Data regarding antibacterial and steroid treatment, blood transfusion, renal replacement therapy, total parenteral nutrition (TPN) and use of invasive devices (a central venous catheter, tracheal tube and urinary tract catheters) were collected.

#### Secondary infections

Secondary infection in septic shock patients was defined as a new infection acquired more than 48 hours after admission to the ICU. The diagnosis of a secondary infection was performed according to the criteria of the Centers for Disease Control and Prevention (CDC, 2008)^34^. Infection was differentially diagnosed from colonization according to the CDC criteria and required one or more new antibiotics^7^,^21^. The time, site and pathogen isolated for the secondary infection were recorded, and the analysis was restricted to the first episode of secondary infection at the same site.

#### Endpoints

Mortality as well as hospital and ICU LOS data were collected.

### Statistical analysis

Data are expressed as percentages or the mean ± SD or interquartile ranges (25^th^ and 75^th^ percentiles). The continuous variables and categorical variables were analyzed with the Mann-Whitney U test or the Chi-square test, respectively. Time trends for total mortality and percentages of early and late death rates were analyzed with Spearman’s rank correlation test. Univariate and multivariate binary logistic regression analyses were used to examine risk factors for secondary infection. To investigate the impact of secondary infection on hospital mortality, univariate analysis was performed to detect the potential variables associated with mortality and variables with a conservative significance level of 0.05 were used for further analysis. We used Spearman rank correlation to assay the correlation between variables. The high correlations (>40%) between SOFA score and SAPS II score, between SAPS II score and age were observed and the SAPS II score was left out of the analysis. Then, the Cox model was used, and secondary infection was modeled as a time-varying covariate by the ‘survival’ package in R^35^,^36^,^37^. Additionally, cumulative incidence functions were calculated by the “cmprsk” package in R to understand how risk accumulates with time^35^,^36^,^37^.

Linear regression analysis was performed to detect the potential variables associated with hospital LOS. Variables with a conservative significance level of 0.05 were used for further analysis. To calculate the difference in length of stay between patients with and without secondary infection, a multistate model using the ‘etm’ package in R was performed^23^,^36^. There are four states in our model including admission (state 0), secondary infection (state 1), discharge alive (state 2) and death (state 3). As time passes, patients with secondary infection move from state 0 into state 1, then into state 2 or state 3, while uninfected patients move from state 0 into state 2 or state 3 (see Supplementary Fig. S3).

The data were prepared and analyzed using SPSS 18.0 and R 3.3.0 software for windows. Statistical significance was expressed as both p values and CI_95_. A two-sided p*-*value < 0.05 was considered statistically significant.

## Additional Information

**How to cite this article**: Zhao, G.-j. *et al*. Incidence, risk factors and impact on outcomes of secondary infection in patients with septic shock: an 8-year retrospective study. *Sci. Rep.* **6**, 38361; doi: 10.1038/srep38361 (2016).

**Publisher's note:** Springer Nature remains neutral with regard to jurisdictional claims in published maps and institutional affiliations.

## Supplementary Material

Supplementary Information

## Figures and Tables

**Table 1:** Clinical baseline characteristics of patients with septic shock.

Parameter	Finding
n	476
Age (years), median (25th, 75th)	64 (53, 75)
Male, n(%)	285 (59.9%)
Comorbidities, n(%)	
Chronic cardiac disease	164 (34.5%)
Diabetes	89 (18.7%)
hepatic cirrhosis	57 (11.9%)
Chronic kidney disease	46 (9.7%)
Cancer or tumor	57 (7.6%)
Chronic pulmonary disease	34 (7.1%)
Admission category, n(%)	
Medical	364 (76.5%)
Surgical	112 (23.5%)
Site of infection, n(%)	
Abdominal	202 (42.4%)
Respiratory tract	123 (25.8%)
Urinary tract	63 (13.2%)
Skin and soft tissue	60 (12.6%)
Others	28 (5.9%)
Positive blood culture, n(%)	
Total	163 (34.2%)
Gram-negative only	94 (56.7%)
Gram-positive only	29 (17.8%)
Fungi only	27 (16.6%)
Polymicrobial	13 (7.9%)
SAPS II at onset of shock, median (25th, 75th)	48 (42, 56)
SOFA score at onset of shock, median (25th, 75th)	10 (7.0, 12)
Length of ICU stay, median (25th, 75th)	6 (3, 11)
Length of hospital stay, median (25th, 75th)	12 (6, 21)
In-ICU mortality, n(%)	195 (41.0%)
In-hospital mortality, n(%)	204 (42.8%)

SAPS II: Simplified Acute Physiology Socre II; SOFA: Sequential Organ Failure Assessment.

**Table 2:** Pathogens identified from patients with secondary infection.

Pathogen	PI (%)	BSI (%)	SSI/STI (%)	UTI (%)	Others (%)
	n = 117	n = 47	n = 27	n = 18	n = 12
A.baumannii	22.2%	6.4%	0	5.6%	8.3%
P. aeruginosa	10.3%	4.3%	25.9%	0	0
C. albicans	8.5%	17.0%	11.1%	44.4%	25.0%
S. maltophilia	8.5%	0%	7.4%	0	0
B. cepacia	7.7%	6.4%	0	0	0
Staphylococcus	6.8%	27.7%	22.2%	5.6%	8.3%
E. coli	4.3%	2.1%	3.7%	22.2%	0
E. faecium	3.4%	12.8%	11.1%	5.6%	16.7%

PI: pulmonary infection; BSI: bloodstream infection; SSI/STI: surgical site and soft tissue infections; UTI: urinary tract infection.

**Table 3:** A comparison of the demographic and clinical characteristics of patients with and without secondary infection.

Variables	Non-SI (n = 227)	SI (n = 145)	Univariate analysis	
			*OR*(*CI*_*95*_)	*P* value
Age (years)				
Median (25th, 75th)	61 (51, 71)	66 (58, 78)		**<0.001**
Age > 65 years, n(%)	83 (36.6%)	78 (53.8%)	2.02 (1.32~3.09)	**0.001**
Male, n(%)	124 (54.6%)	93 (64.1%)	1.49 (0.97~2.28)	0.070
Comorbidities				
≥two comorbidities	42 (18.5%)	36 (24.8%)	1.46 (0.88~2.41)	0.144
Admission category, n(%)				
Medical	194 (85.5%)	111 (75.9%)		
Surgical	33 (14.5%)	34 (24.3%)	1.80 (1.06~3.07)	**0.029**
Immunosupressive agent, n(%)				
Corticosteroid	8 (3.5%)	8 (5.5%)	1.60 (0.57~4.36)	0.355
Other Immunospressive drugs	11 (4.8%)	6 (4.1%)	0.85 (0.31~2.34)	0.750
alcohol abuse	64 (28.2%)	41 (28.3%)	1.00 (0.63~1.60)	0.986
Site of infection, n(%)				
Abdominal	88 (38.8%)	56 (38.6%)	0.99 (0.65~1.53)	0.440
Respiratory tract	59 (26.0%)	43 (29.7%)	1.20 (0.76~1.91)	0.978
Urinary tract	35 (23.1%)	19 (14.3%)	0.83 (0.45~1.51)	0.536
Skin and soft tissue	28 (12.3%)	24 (16.8%)	1.41 (0.78~2.54)	0.253
Positive blood culture, n(%)	81 (35.8%)	51 (35.2%)	0.97 (0.63~1.52)	0.896
SAPS II score at onset of shock				
Median(25th, 75th)	46.0 (39.0, 55.0)	53.00 (45.0, 61.0)		<0.001
SOFA score at onset of shock				
Median (25th, 75th)	9.0 (6.0, 11.0)	10.00 (8.00 14.0)		**<0.001**
Duration of shock, Days				
Median (25th, 75th)	4.0 (3.0, 6.0)	(3.0, 10.0)		**<0.001**
Interventions				
Steroid treatment	88 (38.8%)	85 (58.6%)	2.24 (1.46~3.42)	**<0.001**
blood transfusion	156 (68.7%)	120 (82.8%)	2.19 (1.31~3.83)	**0.003**
Total parenteral nutrition	73 (32.2%)	60 (41.2%)	1.49 (0.97~2.30)	0.070
Renal replacement therapy	27 (11.9%)	36 (24.8%)	2.45 (1.41~4.24)	**0.001**
Intubation	70 (30.8%)	98 (67.6%)	4.68 (2.99~7.32)	**<0.001**
Deep vein catheterization	182 (80.2%)	126 (86.9%)	1.64 (0.92~2.94)	0.094
Length of ICU stay				
Median (25th, 75th)	6 (4, 10)	11 (6, 19)		**<0.001**
ICU mortality, n(%)	58 (25.6%)	62 (42.8%)		**0.001**
Length of hospital stay				
Median (25th, 75th)	13 (8, 20)	19 (11, 33)		**<0.001**
In-hospital mortality, n(%)	59 (26.0%)	69 (47.6%)		**<0.001**
Death due to withdrawal of care, n(%)	12 (20.7%)	15 (24.1%)		0.067

SAPS II: Simplified Acute Physiology Socre II; SOFA: Sequential Organ Failure Assessment; CI_95_: 95% confidence intervals; OR: odds ratio.

**Table 4:** Results of the logistic regression analysis of secondary infection.

Variables	OR	CI_95_	P value
Ages	1.020	1.004~1.036	0.016
SOFA score	1.117	1.040~1.200	0.002
Intubation	2.462	1.492~4.061	<0.001
ICU LOS	1.070	1.036~1.105	<0.001

SOFA: Sequential Organ Failure Assessment; LOS: Length of stay; CI_95_: 95% confidence intervals; OR: odds ratio.

**Table 5:** Results of the Cox-proportional hazard analysis of hospital mortality.

Variables	Unadjusted model	Adjusted model
	HR (95% CI)	HR (95% CI)
Secondary infection	5.675 (3.652~8.819)***	5.607 (3.612~8.704)***
Urinary tract infection	0.322 (0.150~0.689)**	0.311 (0.142~0.680)**
Positive blood culture	1.484 (1.124~1.959)**	1.130 (1.130~1.961)**
Respiratory tract infection	1.514 (1.051~2.180)*	1.140 (0.779~1.671)
SOFA score	1.015 (0.971~1.061)	0.999 (0.952~1.047)
Duration of shock (day)	1.005 (0.988~1.022)	0.999 (0.982~1.016)
Age > 65 years	1.672(1.170~2.388)**	1.588 (1.098~2.295) *

HR: Hazard Ratio; SAPS II: Simplified Acute Physiology Socre II; CI: confidence intervals; *P < 0.05; **P < 0.01; ***P < 0.001.
