# Supplementary material for: Incidence, risk factors and impact on outcomes of secondary infection in patients with septic shock: an 8-year retrospective study
Source: Sci Rep. 2016 Dec 7;6:38361. doi: 10.1038/srep38361 (PMC5141415; doi:10.1038/srep38361)
Supplement: Supplementary Information [file srep38361-s1.pdf]

Incidence, risk factors and impact on outcomes of secondary infection in patients with septic shock: an 8-year retrospective study

Guang-ju ZHAO, Dong LI, Qian ZHAO, Jia-xing SONG, Xiao-rong CHEN,
Guang-liang HONG, Meng-fang LI, Bing WU, Zhong-qiu LU

Supporting information:

Figure S1: Time distribution of septic shock deaths

Figure S2: The frequency of secondary infection over time

Figure S3: Multistate model used to determine hospital length of stay

Table S1: Demographic and clinical characteristics of survivors and non-survivors.

Table S2: The effect of patient characteristics on hospital length of stay by univariate analysis.

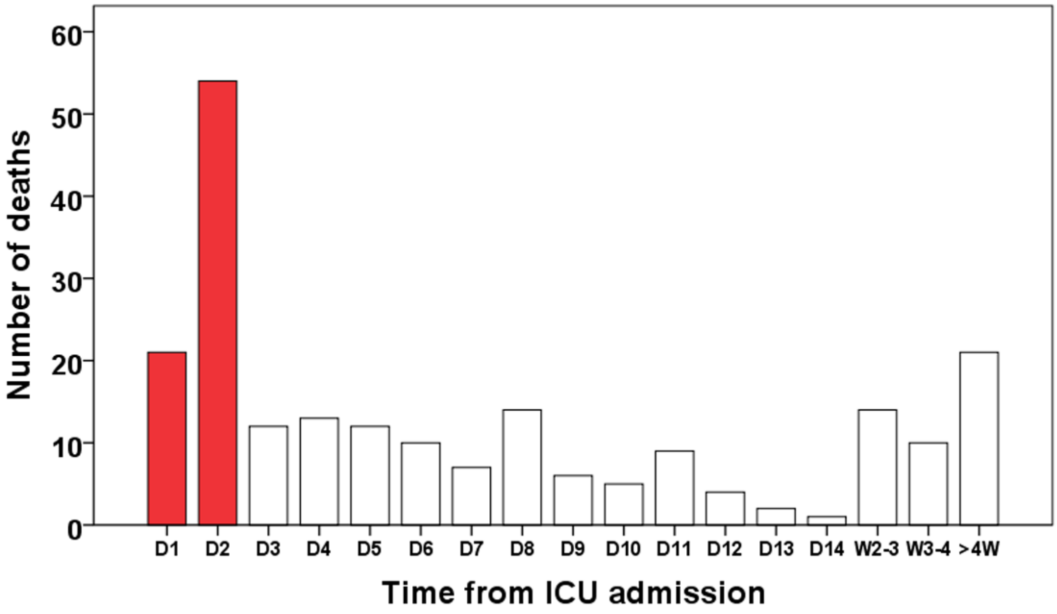

Figure S1. Time distribution of septic shock deaths

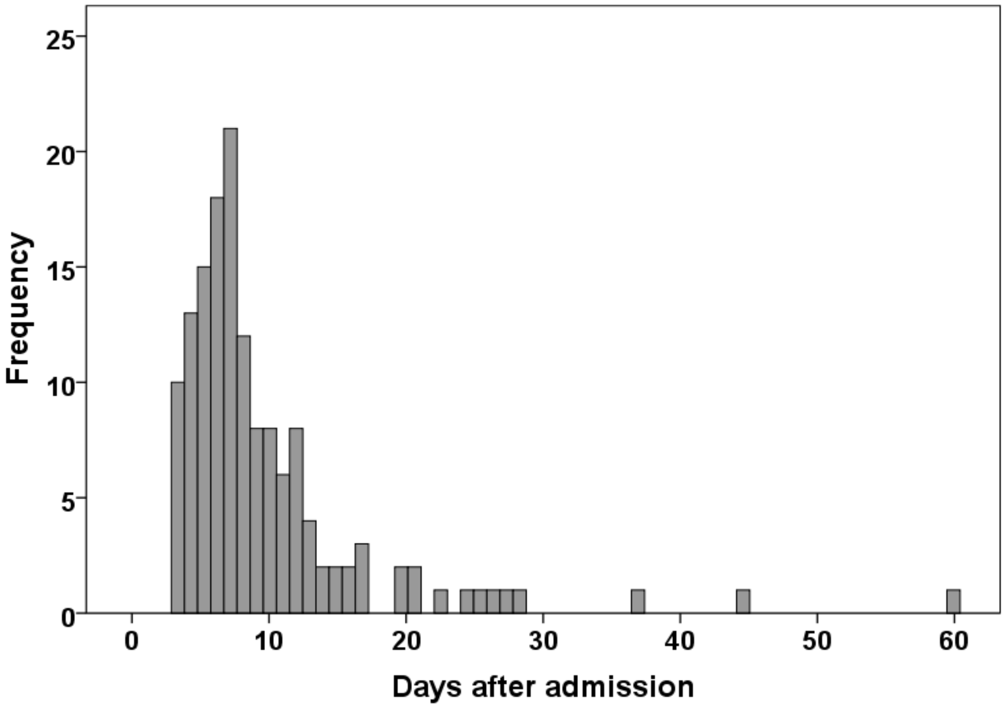

Figure S2: The frequency of secondary infection over time

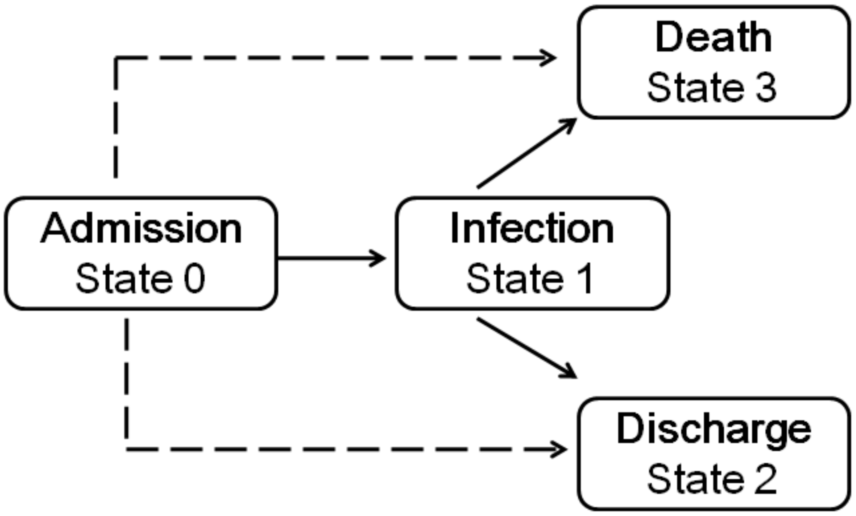

Figure S3. Multistate model used to determine hospital length of stay

Table S1. Demographics and clinical characteristic of late-phase septic shock patients

**Variables**	**Total (n=372)**	**Survivors (n=244)**	**Non-Survivors (n=128)**	***P* value**
**Age (years)**				
Median (25th, 75th)	63 (52, 75)	60 (50, 70)	70 (58, 80)	< **0.001**
**>65 years,** n(%)	161 (43.3%)	87 (35.7%)	74 (57.8%)	< **0.001**
**Male,** n(%)	217(58.3%)	135 (55.7%)	82 (64.1%)	0.121
**Comorbidities,** n(%)				
Chronic cardiac disease	135 (36.3%)	80 (32.8%)	55 (43.0%)	0.052
Chronic pulmonary disease	28 (7.5%)	15 (6.15%)	13 (10.2%)	0.164
Kidney failure	32 (8.6%)	21 (8.6%)	11 (8.6%)	0.997
hepatic cirrhosis	34 (9.1%)	23 (9.4%)	11 (8.6%)	0.791
Diabetes	75 (20.2%)	55 (22.5%)	22 (17.2%)	0.226
Cancer or tumor	37 (9.9%)	23 (9.4%)	14 (10.9%)	0.852
**Admission category,** n(%)				
Medical needs	306 (82.3%)	200 (82.0%)	106 (82.8%)	
Surgical needs	66 (17.7%)	44 (28.0%)	22 (27.2%)	0.839
**Immunosupressive agent,** n(%)				
Corticosteroid	16 (4.3%)	8 (3.3%)	8 (6.3%)	0.180
Other immunospressive drugs	17 (4.6%)	11 (4.5%)	6 (4.7%)	0.937
Alcohol abuse	105 (28.2%)	70 (28.7%)	35 (27.3%)	0.784
**Site of infection,** n(%)				
Respiratory tract	101 (27.2%)	57 (23.3%)	44 (34.3%)	**0.023**
Urinary tract	54 (14.5%)	46 (18.9%)	8 (6.3%)	< **0.001**
Abdominal	145 (38.9%)	91 (37.3%)	54 (42.2%)	0.358

Skin and soft tissue	52 (14.0%)	34 (13.9%)	18 (14.1%)	0.973
**Positive blood culture,** n(%)	141 (37.9%)	78 (32.1%)	63 (49.2%)	**0.001**
**SAPS II at onset of shock**				
Overall, median (25th, 75th)	48 (41, 57)	46 (38, 54)	55 (47, 64)	**< 0.001**
**SOFA score at onset of shock**				
Overall, median (25th, 75th)	8 (10, 12)	9.5 (7, 12)	10 (8, 13)	**0.011**
**Duration of shock,** days	4 (3, 8)	4 (3, 7)	5 (3, 9)	**0.018**
**Secondary infection**, n(%)	128 (39.0%)	76 (31.1%)	69 (47.6%)	**< 0.001**

SAPS II: Simplified Acute Physiology Socre II; SOFA: Sequential Organ Failure Assessment

Table S2. The effect of patient characteristics on hospital LOS by univariate analysis

Variable	Median (25th, 75th) LOS (days)		*P* value
	Yes	No	
**Age>65 years**	13 (8, 28)	15 (10, 23)	0.596
**Male**	14 (9, 27)	15 (10, 22)	0.515
**≥ two comorbidities**	14 (9, 27)	14 (9, 23)	0.602
**Immunosuppressive agents**			
alcohol abuse	17 (10, 28)	14 (9, 22)	**0.019**
Corticosteroid	14.5 (8, 27)	14 (9, 24)	0.846
Other immunosuppressive drugs	15 (8, 27)	14 (9, 24)	0.939
**Surgical needs**	18 (9, 31)	14 (9, 23)	0.139
**Site of infection**			
Abdominal	13 (9, 21)	15 (9, 26)	0.063
Respiratory tract	14 (9, 24)	14 (9, 25)	0.957
Urinary tract	16 (12, 25)	14 (9, 24)	0.185
Skin and soft tissue	22 (9, 32)	14 (9, 22)	**0.046**
**Positive blood culture**	14 (8, 26)	14 (10, 23)	0.562
**SOFA score**			
<8	13 (10, 21)	15 (9, 26)	0.286
8-12	13 (8, 22)	15 (11, 27)	0.057
>12	19 (11, 31)	13 (9, 22)	**0.001**
**SAPS II score**			
<41	14 (10, 23)	14 (7, 29)	0.221
41-56	15 (10, 23)	13 (7, 25)	0.188
>56	13 (9, 22)	14 (10, 25)	0.721
**Duration of shock**			
>7 days	19.5 (14, 29)	13 (8, 22)	**<0.001**
4-7 days	12 (8, 19)	17 (11, 26)	**<0.001**
<4 days	13.5 (9, 25)	14 (9, 25)	0.333
**Interventions**			
Steroid treatment	17 (10, 29)	13 (9, 21)	**0.001**
blood transfusion	15 (9, 27)	13 (10, 19)	0.144
Total parenteral nutrition	14 (8, 23)	14 (9, 25)	0.466
Renal replacement therapy	16 (8, 29)	14 (10, 23)	0.700
Intubation	16 (7, 30)	13 (10, 21)	0.096
Deep vein catheterization	15 (9, 26)	12 (8.25 19)	**0.023**
**Secondary infection**	19 (11, 33)	13 (8, 20)	**<0.001**

SAPS II: Simplified Acute Physiology Socre II; SOFA: Sequential Organ Failure Assessment; LOS, length of stay

Columns "Yes" and "No" refer to the presence and absence of the variables in the "Variable" column, respectively.
